# Identifying metabolism-related genes in liver cancer through weighted gene co-expression network analysis and machine learning

**DOI:** 10.3389/fgene.2025.1654459

**Published:** 2025-09-24

**Authors:** Taorui Wang, Zijun Lai, Shengjun Tang, Lehang Lin, Mingjiao Zhang

**Affiliations:** ^1^ Faculty of Medicine, Macau University of Science and Technology, Taipa, China; ^2^ Genetic Testing Center, Guangzhou Women and Children’s Medical Center, Guangzhou Medical University, Guangzhou, China; ^3^ Guangdong Provincial Key Laboratory of Malignant Tumor Epigenetics and Gene Regulation, Guangdong-Hong Kong Joint Laboratory for RNA Medicine, Medical Research Center, Sun Yat-Sen Memorial Hospital, Sun Yat-Sen University, Guangzhou, China; ^4^ Department of Rheumatology and Immunology, the Third Affiliated Hospital of Southern Medical University, Guangzhou, China

**Keywords:** liver cancer, metabolism, machine learning, immune cells, therapy

## Abstract

**Objective:**

As a leading cause of cancer-related mortality, liver cancer was associated with metabolic dysregulation. We aimed to identify metabolism-related prognostic biomarkers and therapeutic targets.

**Methods:**

Transcriptomic data from TCGA were analyzed using EdgeR to identify differentially expressed genes (DEGs). WGCNA was applied to unveil the metabolism-related genes in liver cancer. Machine learning algorithms (RF, SVM, LASSO) refined marker genes. GSEA and ssGSEA were conducted to identify pathway associations and immune interactions of marker genes. DGIdb database predicted candidate therapeutics targeting these biomarkers. The independent queue (GSE54236) was verified as an external dataset. RT-PCR validated gene expression in clinical samples.

**Results:**

A total of 234 metabolism-related genes were identified in liver cancer. Through undergoing machine learning by RF, SVM, and LASSO algorithms, seven marker genes (ACADS, ALDH8A1, COX4I2, CYP2C8, DBH, NDST3, and PLA2G6) were obtained. Except for PLA2G6, the other genes were correlated with the survival of patients with liver cancer and immune cells infiltration. Additionally, ACADS, ALDH8A1, CYP2C8, DBH, and NDST3 were downregulated, and COX4I2 was upregulated in dataset of GSE54236, which were consist with those in TCGA database. However, RT-PCR validation in 10 paired clinical samples confirmed significant downregulation of ACADS, ALDH8A1, COX4I2, CYP2C8, DBH, and NDST3 in tumor tissues (all P < 0.05). Immune infiltration analysis revealed these genes might influence immune cell infiltration in the tumor microenvironment. And the candidate drugs were unveiled, including PAZOPANIB, SUMATRIPTAN, ETOPOSIDE, etc.

**Conclusion:**

The metabolism-related biomarkers ACADS, ALDH8A1, COX4I2, CYP2C8, DBH, and NDST3 demonstrated significant potential for predicting liver cancer prognosis and may serve as candidate therapeutic targets.

## Introduction

Liver cancer is the top 5 leading cause of death related to cancer, having affected 905,700 individuals worldwide in 2020. It is predicted to affect 1.4 million persons by 2040 (Rumgay et al., 2022). Recent data reveal that the 5-year relative survival rate for liver cancer remains alarmingly low at approximately 20%, with only marginal improvements over the past decade (Tapper and Parikh, 2023; Chen et al., 2024). The risk factors for liver cancer include viral hepatitis, alcohol, and cirrhosis, etc. (Konyn et al., 2021). Even though surgery is the primary treatment for early-stage liver cancer, postoperative mortality ranges from 20% to 40% (Maki and Hasegawa, 2022). In addition, most patients are diagnosed at end-stage, where tyrosine kinase inhibitors (TKIs) are applicable. However, TKIs are available for only 30% of patients and the survival is slightly improved (Chidambaranathan-Reghupaty et al., 2021). It has been reported that immunosuppression, metabolic alterations, and inflammation, etc. contribute to the progression of liver cancer (Li et al., 2021). However, it is not fully understood. Further investigation of the underlying mechanisms is valuable to identify candidate therapeutic targets against liver cancer.

Metabolic reprogramming is recognized as a key hallmark of hepatocellular carcinoma (HCC), and it plays a crucial role in promoting cancer cell proliferation and survival (Bergers and Fendt, 2021). Alterations in metabolic pathways, including glycolysis (Zhang D. et al., 2019), lipid metabolism (Wu et al., 2024), and amino acid catabolism (Mossmann et al., 2023), enable tumor cells to meet the high demands of biosynthesis and energy production. In HCC, increased glycolytic flux (the Warburg effect) and changes in mitochondrial function facilitate tumor growth under both normoxic and hypoxic conditions (Paul et al., 2022). Key regulatory enzymes such as pyruvate kinase M2 (PKM2) and lactate dehydrogenase A (LDHA) have been identified as critical factors in driving HCC progression. Additionally, the accumulation of reactive oxygen species (ROS) and the altered balance of oxidative phosphorylation further contribute to genomic instability and tumor metastasis. Critical genes involved in the dysregulation of these metabolic pathways, for instance, ALDH8A1 (Aldehyde Dehydrogenase 1 Family, Member A1) is involved in detoxifying aldehydes, with its altered expression linked to tumor progression and chemotherapy resistance (He et al., 2020). COX4I2 (Cytochrome C Oxidase Subunit 4, Isoform 1), part of the mitochondrial electron transport chain, is vital for oxidative phosphorylation, and its dysfunction is associated with increased ROS production in HCC (Xue et al., 2023). These metabolic genes contribute to the complex metabolic phenotype of HCC and offer valuable insights into potential therapeutic targets. Understanding how these genes interact can pave the way for new treatment strategies and diagnostic approaches, emphasizing the importance of metabolic interventions in HCC therapy.

Machine learning offers an efficient approach to identify the genes that have critical roles in the progression of disease. Some algorithms such as Least Absolute Shrinkage and Selection Operator (LASSO), Support Vector Machine (SVM), and RandomForest are usually used to carry out the machine learning. In addition, the Weighted Gene Co-expression Network Analysis (WGCNA) can identify hub genes which are significantly correlated with the disease. Hence, these methods accelerate discovery of key genes associated with disease. Previously, machine learning has been used to seek out the biomarkers in liver cancer (Lv et al., 2023). However, no study has identified metabolism-related marker genes in liver cancer using machine learning. In our study, we aimed to perform machine learning to identify the metabolism-related marker genes in liver cancer. A total of 7 genes (ACADS, ALDH8A1, COX4I2, CYP2C8, DBH, NDST3, and PLA2G6) were obtained. By investigating their expressions and correlations with survival, as well as unveiling the related pathways and their associations with immune cells infiltration, the potential roles of these metabolism-related marker genes were uncovered.

## Materials and methods

### Data collection and processing

The transcriptional profiles of liver cancer were downloaded from the TCGA database, including 374 samples with liver cancer and 50 normal samples. The raw data were converted into an expression matrix, which was normalized with transcripts per million (TPM). The dataset of GSE54236 was obtained from the Gene Expression Omnibus database, comprising 81 tumor samples and 89 adjacent normal tissue samples. Raw expression data underwent log2 transformation followed by quantile normalization to minimize technical variations between samples. Probes were mapped to gene symbols, and duplicate gene entries were resolved by retaining the probe with the highest mean expression intensity per gene, yielding unique gene-level expression profiles. Normalized expression data were subjected to Student’s t-tests to identify differentially expressed genes (DEGs) between tumor and normal groups. Genes with an absolute log2 fold change (FC) > 1 and p-value <0.05 were considered statistically significant. Receiver Operating Characteristic (ROC) analysis was performed using GraphPad Prism software. And the area under the ROC curve (AUC) was used to indicate the diagnostic potential for distinguishing tumor from normal tissues. A total of 5871 metabolism-related genes were obtained from the KOBAS software using the keyword “metabolic pathways”.

### Identification of differentially expressed genes (DEGs) in liver cancer

The “edgeR” (3.42.4) package in R (4.3.1) was used to identify the DEGs in liver cancer by setting the criteria as *p* < 0.05 and |log2FC| >1. And statistical significance was defined as a false discovery rate (FDR) < 0.05.

### Functional enrichment analyses

The gene ontology (GO) and Kyoto Encyclopedia of Genes and Genomes (KEGG) enrichment analyses were performed using the KOBA software by setting the *p-*value less than 0.05.

### Weighted gene co-expression network analysis (WGCNA)

Gene co-expression networks were constructed using WGCNA (v1.72-1) in R (4.3.1) to identify modules of co-expressed genes and their associations with cancer phenotype (Xu et al., 2023). Initially, an appropriate soft thresholding power (β) was determined to transform the gene correlation matrix into a weighted adjacency matrix, adhering to scale-free topology criteria (selecting the smallest β where the scale-free topology model fitted R^2^ exceeded 0.9) to approximate a scale-free network while preserving connectivity information. Using this β, the adjacency matrix was calculated and subsequently transformed into a Topological Overlap Matrix (TOM) to account for shared neighbors. Genes were then hierarchically clustered based on TOM dissimilarity, and dynamic tree cutting with a merging threshold (set to 0.4) groups closely related branches into distinct modules, each represented by a unique color and potentially sharing functional coherence. The module eigengene (ME), representing the first principal component of a module’s gene expression, was extracted for each module. Module-trait association analysis assessed the correlation (using Pearson’s correlation coefficient) and its significance (p-value) between each ME and the binary phenotypic trait of cancer status (disease vs. control). Modules demonstrating significant association (p-value <0.05) with cancer status were prioritized for further investigation. Relationships between modules were also analyzed via eigengene network analysis. Within the cancer-associated modules, hub genes functionally linked to the module’s behavior and the trait were identified based on high module membership (MM, correlation between a gene’s expression and the module eigengene, MM > 0.6) and high gene significance (GS, absolute correlation between the gene’s expression and the cancer trait, GS > 0.5), resulting in a set of candidate genes potentially related to cancer pathogenesis.

### Identification of metabolism-related DEGs in liver cancer

The DEGs of liver cancer, hub genes identified by the WGCNA, and the metabolism-related genes obtained from the KOBAS software were used for the Venn analysis. The intersecting genes were considered as the metabolism-related DEGs in liver cancer.

### Machine learning algorithms

To identify crucial genes associated with metabolic alterations in cancer, we used three machine learning algorithms: Random Forest (RF), Support Vector Machine (SVM), and Least Absolute Shrinkage and Selection Operator (LASSO) regression. These models were applied to the 234 metabolism-related genes obtained from previous differential analysis and WGCNA. And a total of 424 samples were randomly split into training and test sets in a 3:7 ratio. All models were implemented in R (4.3.1) with 10-fold cross-validation to ensure robustness. The intersection of genes selected by the three models served as marker genes for liver cancer onset, aiming to identify the key genes that can truly distinguish cancer patients from healthy individuals.

RF was performed using the RandomForest package. A 10-fold cross-validation was applied were used to ensure robust model performance. Feature genes were selected based on the following criteria: MeanDecreaseAccuracy >0 (improvement in classification accuracy) and MeanDecreaseGini >0 (contribution to node purity), which indicates the importance of each gene in differentiating the two groups. Model performance was internally validated through out-of-bag (OOB) error estimation during the 10-fold CV process. The model parameters were set as: method = “rf”, summaryFunction = “twoClassSummary”, and tuneLength = “10”.

The “kernlab” package was employed to implement the SVM. A 10-fold cross-validation approach was used. Linear and RBF kernels was tested by varying loss function parameters in a range from 0 to 1 to train the model. Feature genes were selected based on their performance in the model, ensuring that only those consistently contributed to classification accuracy across CV folds were retained. The model parameters were set as: method = “svmRadial”, summaryFunction = “twoClassSummary”, Cost = “seq (0.1, 2)”, length = “10”, and sigma = “0.1”.

For LASSO regression, the “glmnet” and “foreign” R packages were used. cv.glmnet with binomial family for binary classification was employed for training. Features with non-zero coefficients were selected as the final set of important genes, which were used for further analysis and model development. The model parameters were set as: Family = “binomial”, alpha = “1”, type.measure = “class”, and nfolds = “10”.

### Gene set enrichment analysis (GSEA)

GSEA was performed using the “clusterProfiler” package to assess the enrichment of marker genes in predefined gene sets. The background datasets utilized in this analysis were derived from the Gene Ontology (GO, 2018) and Kyoto Encyclopedia of Genes and Genomes (KEGG, 2019) collections. The analysis was conducted in three major steps: (1) Calculation of Enrichment Score (ES), the ES for each gene set was calculated by ranking all genes in the dataset based on their correlation with the phenotype of interest. (2) Estimation of Significance of Enrichment Score, to determine the statistical significance of the observed ES, a permutation-based approach was applied. Gene set enrichment scores were computed for a large number of random gene set permutations, generating a null distribution of ES values. The empirical *P* was then derived by comparing the observed ES to the null distribution. A lower *P* indicates a more significant enrichment of the gene set. (3) Multiple Hypothesis Testing, given the large number of gene sets tested, multiple hypothesis testing correction was applied using the Benjamini-Hochberg (BH) method to control the False Discovery Rate (FDR). Gene sets with an FDR-adjusted *P* < 0.05 were considered significantly enriched. This adjustment ensures that the analysis accounts for the inherent multiple testing burden, minimizing the likelihood of false-positive findings.

### Survival

Survival analysis of marker genes was conducted based on their expression levels. The expression levels of marker genes were categorized into two groups: high and low expression. The high expression group consisted of samples with expression levels above the median, while the low expression group included samples with expression levels below the median. For survival analysis, the marker gene expression levels were correlated with the survival time of cancer patients. Kaplan-Meier survival curves were generated to evaluate the association between marker gene expression and patient survival outcomes. The hazard ratios (HR) and log-rank *P* were calculated to assess the impact of gene expression on overall survival. The analysis was performed using the survival package in R, with the survfit function employed to fit the survival model. The resulting survival curves were visualized using the ggsurvplot function from the survminer R package, which provides a graphical representation of the survival data.

### Patients and sample processing

Clinical specimens comprising cancerous tissues (n = 10) and histologically normal hepatic tissues (n = 10) were acquired from 10 patients with liver cancer in Sun Yat-Sen Memorial Hospital following diagnostic confirmation. All samples were collected before therapeutic interventions. Specimens underwent immediate cryopreservation through liquid nitrogen immersion followed by long-term storage at −80 °C until experimental processing. The patients exclusively consisted of HBV-positive individuals with no recorded alcohol usage history, exhibiting tumor progression stages between II and IIIC. This investigation received ethical validation from the Institutional Review Board of Sun Yat-Sen Memorial Hospital (Authorization Code: SYSKY-2023-010-01). And the informed consent form was obtained from all patients. And all methods were performed in accordance with the guidelines and regulations of the Institutional Review Board of Sun Yat-Sen Memorial Hospital.

### RNA extraction and quantitative real-time PCR

Total RNA isolation was performed employing TRIzol reagent (R21086, Yuanye, Shanghai, China) following the manufacturer’s protocol. RNA quantification was conducted via spectrophotometric analysis using a Nanodrop 2000 system (Invitrogen). For cDNA synthesis, 1 μg of purified RNA underwent reverse transcription with the ABScript Neo RT MasterMix for qPCR with the gDNA Remover Kit (RK20433, ABclonal, Wuhan, China). RT-PCR assay was performed using the ChamQ SYBR qPCR Master Mix (Q311-02, Vazyme, Nanjing, China) on a CFX96 Real-Time PCR system (Bio-Rad) with triplicate technical replicates per sample. Primer sequences are shown in Supplementary Table S1.

### Immune cell infiltration analysis

Immune infiltration analysis was performed using the ssGSEA (single-sample Gene Set Enrichment Analysis) method to compute the enrichment scores of each immune cell type for every cancer sample. The IOBR R package was employed, specifically the calculate_sig_score function, to calculate the enrichment scores based on predefined immune cell marker gene sets. The ssGSEA method quantifies the relative abundance of each immune cell type in individual samples, reflecting immune cell infiltration levels. Subsequently, the correlation between marker gene expression and immune cell infiltration was analyzed. The linkET R package, particularly the correlate function, was used to compute the Pearson correlation coefficients between the expression levels of marker genes and the enrichment scores of various immune cell types. This step enabled the identification of significant associations between marker gene expression and immune cell infiltration in the tumor microenvironment. Finally, the correlation between target gene expression and immune cell infiltration was examined. The expression levels of target genes across different cancer samples were correlated with the ssGSEA enrichment scores for each immune cell type, revealing the relationship between the target gene and immune cell populations in the cancer microenvironment.

### Identification of the candidate drugs

To identify candidate drugs targeting marker genes, a list of drugs associated with target transcription factors (TF) and target genes (TG) was obtained from the DGIdb v4.0 database. The database was queried for drugs that interact with the TF-TG pairs corresponding to the identified marker genes. Two drug-gene interaction networks were constructed: one depicting the interaction between drugs and target genes (drug-TG) and another between drugs and transcription factors (drug-TF). The gene-gene-drug interaction network was visualized to identify potential drugs that could target the marker genes.

### Statistical analysis

EdgeR conducts differential expression analysis using generalized linear models, Bayesian estimation, and correction of multiple hypothesis testing. WGCNA analysis utilizes correlation analysis, hierarchical clustering analysis algorithm for correlation, and network partitioning algorithm. RF, SVM, and LASSO regression are used in machine learning. Gene set enrichment analysis and immune cell infiltration analysis are essentially based on the calculation of enrichment scores (ES) and permutation testing algorithms. Survival analysis is based on Kaplan-Meier method and Cox proportional hazards model.

## Results

### Identification of the DEGs in liver cancer

In order to identify metabolism-related DEGs in liver cancer, we first unveiled DEGs in liver cancer by analyzing the data from the TCGA database. After processing the data by the “edgeR” package of R, a total of 4296 DEGs were obtained. Among them, 3312 DEGs were upregulated, and 984 DEGs were downregulated (Supplementary Figure S1A; Supplementary Table S2), which were displayed as volcano plot (Supplementary Figure S1B) and heat map (Supplementary Figure S1C). As shown in Supplementary Figure S1D, the expression profiles between tumor samples and normal samples were obviously grouped, indicating there was a significant difference in gene expression between the two groups. Then we further investigated the associated pathways of these DEGs. The results of GO enrichment analysis indicated these genes were most related to collagen-containing extracellular matrix, cell adhesion, and integral component of plasma membrane, etc. (Supplementary Figure S1E). And signal transduction, cell adhesion, and positive regulation of transcription by RNA polymerase II, etc. were enriched in the biological process. The plasma membrane, integral component of the plasma membrane, and the extracellular space were the most relevant cellular components. For the molecular function, protein binding, calcium ion binding, and identical protein binding were active (Supplementary Figure S1F). And the results of KEGG analysis revealed these DEGs were associated with metabolic pathways, pathways in cancer, and PI3K-Akt signaling pathway, ect. (Supplementary Figure S1G).

### Identification of the metabolism-related DEGs in liver cancer

**FIGURE 1:**
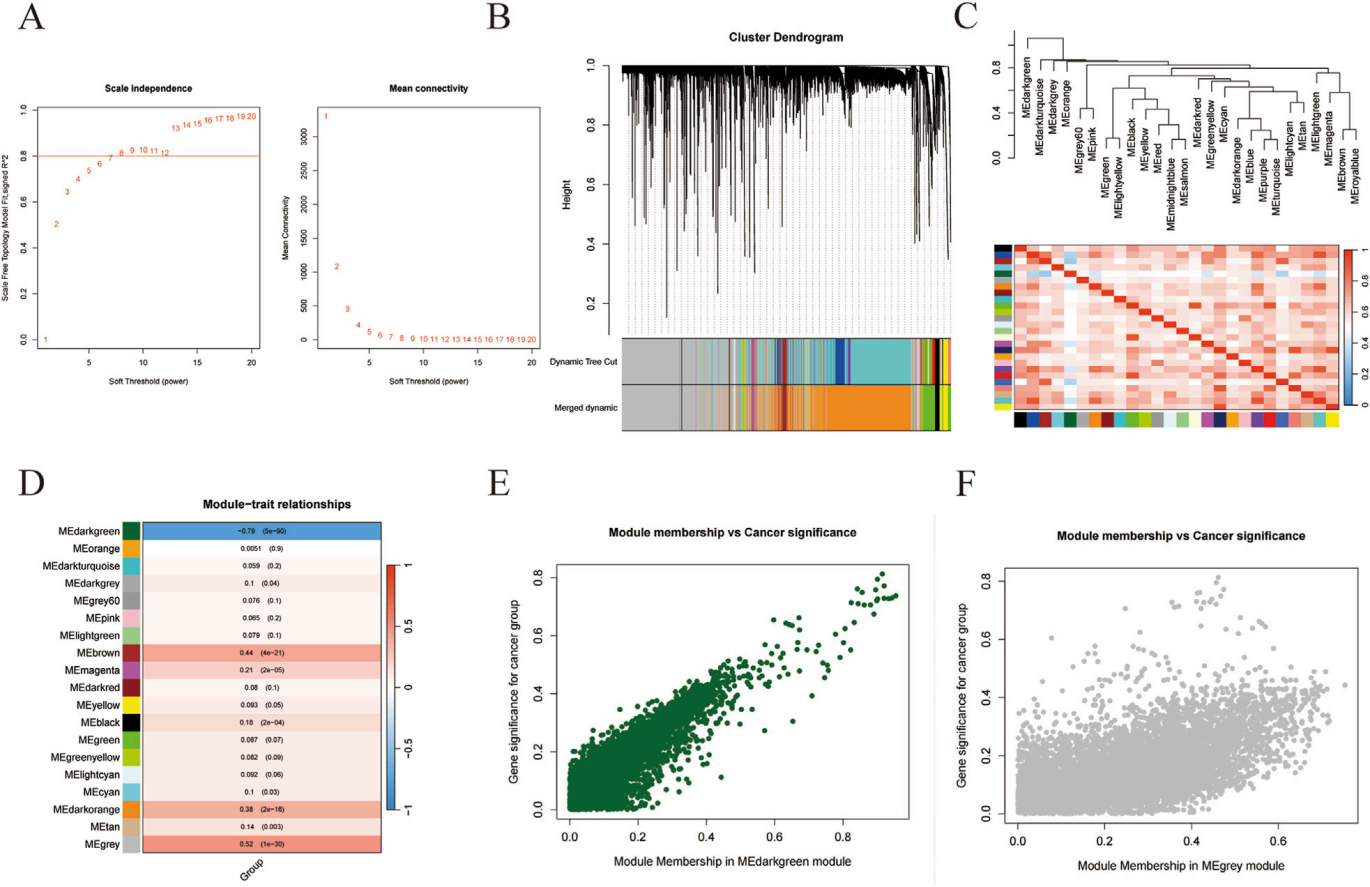
WGCNA analysis of gene in liver cancer. **(A)** The correlation of soft threshold value with scale independence and mean connectivity. **(B)** The cluster dendrogram of modules. **(C)** The similarity between modules. **(D)** The correlations of modules with the trait of samples. **(E,F)** The correlations between cancer significance and module membership in MEdarkgreen **(E)** or MEgray module **(F)**.

Subsequently, we attempted to find out the DEGs that were most correlated with the progress of liver cancer by using WGCNA. As shown in Figure 1A, the co-expression networks of DEGs were closer to a scale-free network when the soft threshold power was set as 8. Then all of the co-expression genes were divided into 19 modules (Figure 1B). And the similarity between different modules was revealed according to the expression of feature genes in the module (Figure 1C). Interestingly, the “MEdarkgreen” module and the “Megrey” module were significantly correlated with the trait (tumor or normal) of samples (Figure 1D). And the correlations between module membership in both modules and cancer significance were investigated, indicating these two modules were positively related to liver cancer (Figures 1E,F). The hub DEGs in the “MEdarkgreen” module (80) and the “Megrey” module (6843) were shown in Supplementary Table S3.

**FIGURE 2:**
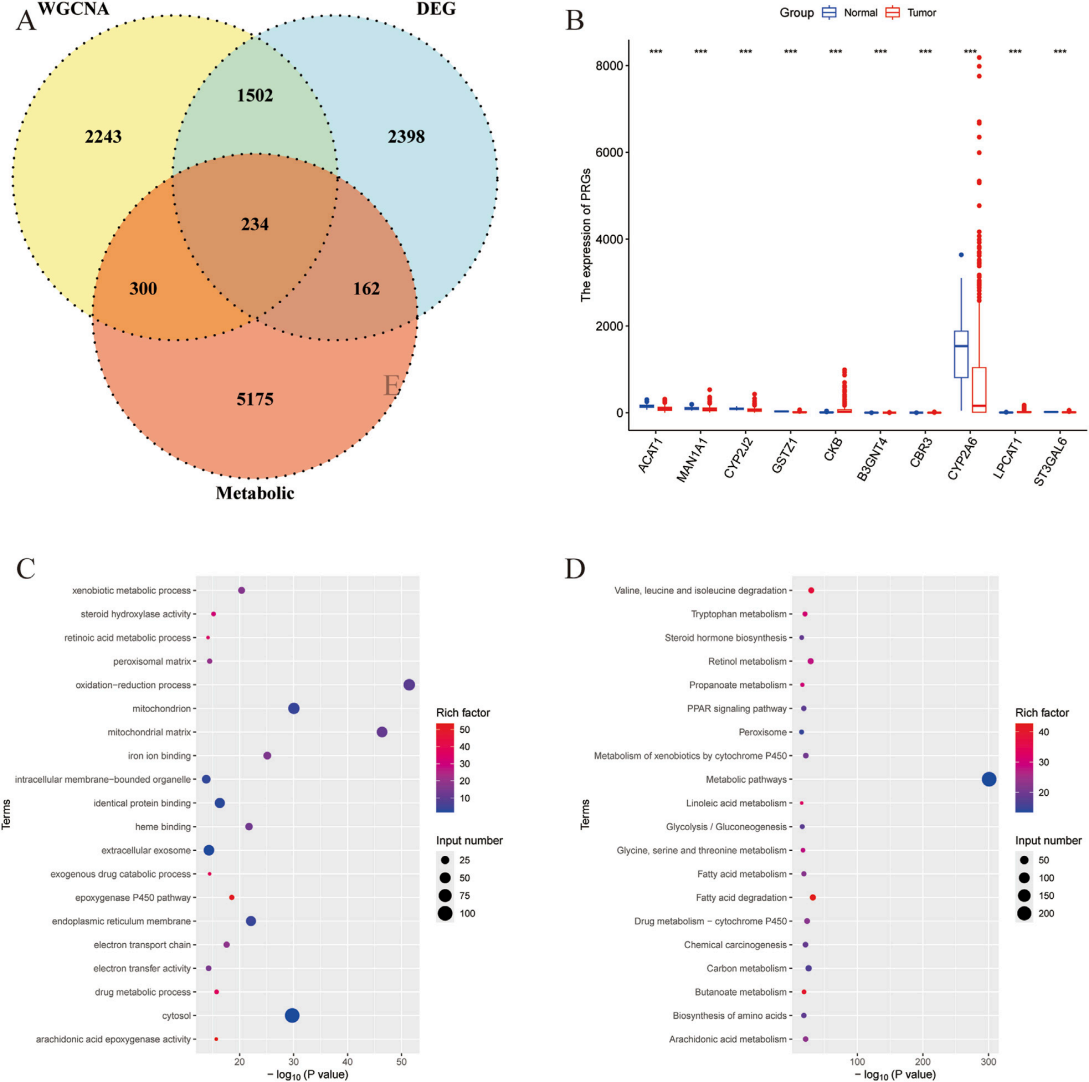
Identification of the metabolism-related DEGs in liver cancer. **(A)** Venn analysis of DEGs of liver cancer, hub genes identified by the WGCNA, and the metabolism-related genes. **(B)** The expression of randomly selected 10 genes. **(C)** GO analysis of the metabolism-related DEGs. **(D)** KEGG analysis of the metabolism-related DEGs.

Then the intersection of DEGs in liver cancer, hub DEGs identified by the WGCNA, and the metabolism-related genes obtained from the KOBAS software were used for the venn analysis. As shown in Figure 2A, a total of 234 DEGs were obtained and considered as metabolism-related DEGs in liver cancer (Supplementary Table S4). The expressions of 10 randomly selected DEGs were revealed in Figure 2B. Additionally, GO and KEGG analyses were performed to unveil the related pathways of the metabolism-related DEGs, indicating that the oxidation-reduction process, mitochondrial matrix, and mitochondrion were most enriched in GO analysis (Figure 2C). And metabolic pathways, fatty acid degradation, and valine, leucine, and isoleucine degradation, etc., were closely associated with the metabolism-related DEGs (Figure 2D).

### Metabolism-related marker DEGs in liver cancer were revealed by machine learning

**FIGURE 3:**
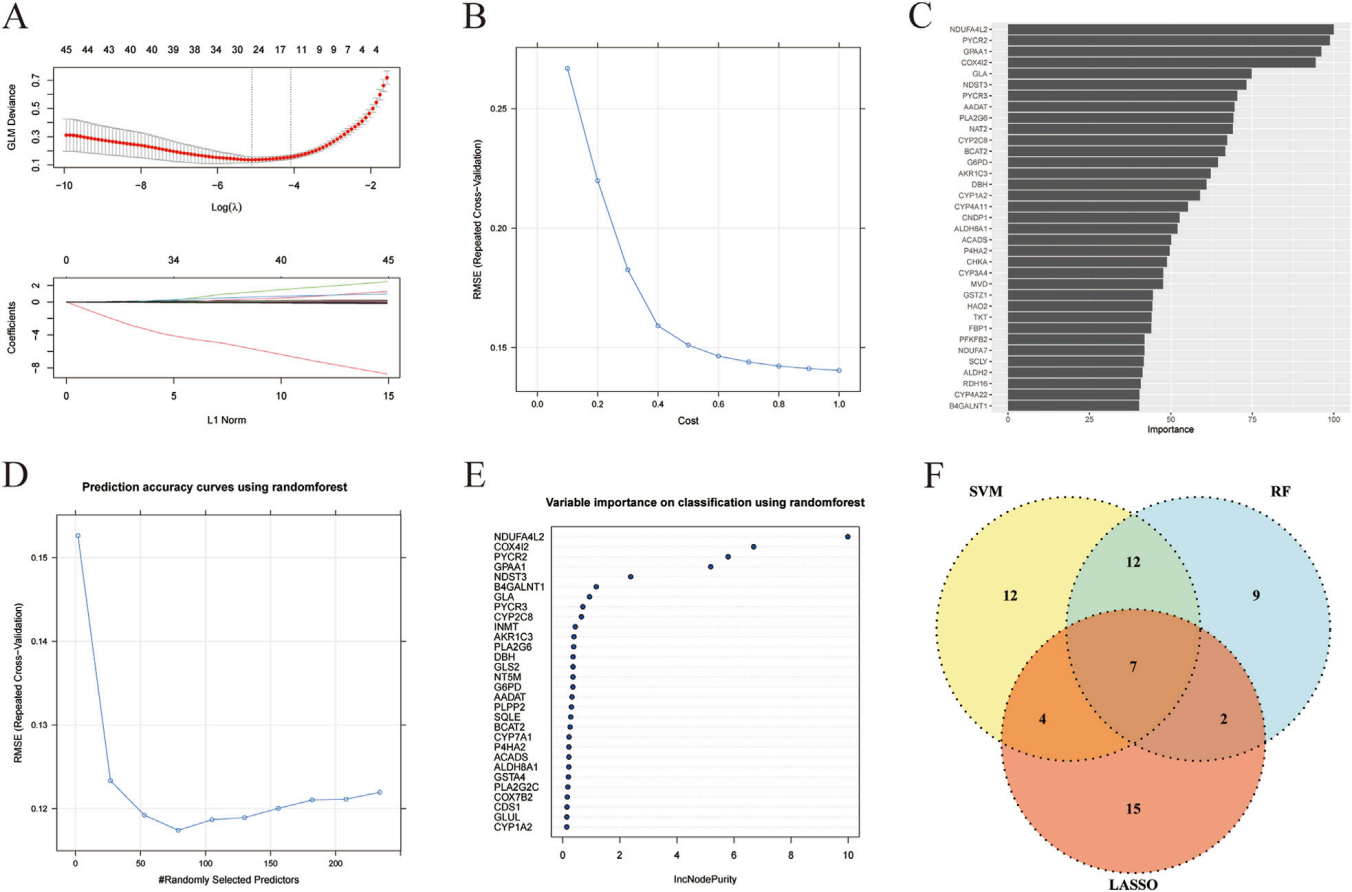
Metabolism-related marker DEGs in liver cancer were revealed by machine learning. **(A)** The correlation of log(λ) and GLM deviance and the correlation of L1 Norm and coefficients in LASSO algorithm. **(B)** The correlation of cost and RMSE in SVM algorithm. **(C)** The importance of feature genes in SVM algorithm. **(D)** The correlation of randomly selected predictors and RMSE in RF algorithm. **(E)** The importance of feature genes in RF algorithm. **(F)** Venn analysis of feature genes obtained from LASSO, SVM, and RF algorithms.

In order to identify the metabolism-related marker DEGs in liver cancer, we then analyzed the 234 metabolism-related DEGs by three machine learning algorithms, including LASSO, SVM, and RF. As shown in Figure 3A, when setting the logλ value as −5 and L1 Nrom as 15, a total of 28 feature genes were obtained by LASSO. Besides, the SVM algorithm revealed that after 10-fold cross-validation, the RMSE would be the smallest when the cost was set as 1.0. And 35 feature genes were obtained (Figure 3B) and their importance was shown in Figure 3C. In addition, the result of RF algorithm showed that when the number of feature genes was 75, the RMSE value would be the smallest (Figure 3D). And the metrics such as accuracy, sensitivity, specificity, precision, F1, and AUC of the three models were shown in Supplementary Table S5. The importance of the top 30 feature genes obtained by RF algorithm was unveiled in Figure 3E. The top 30 feature genes were intersected with the feature genes derived from LASSO and SVM algorithms, a total of 7 feature genes were finally obtained, including ACADS, ALDH8A1, COX4I2, CYP2C8, DBH, NDST3, and PLA2G6 (Figure 3F). And these genes were considered as the metabolism-related marker DEGs in liver cancer.

### Expression profiles and prognostic implications of seven marker genes in liver cancer

**FIGURE 4:**
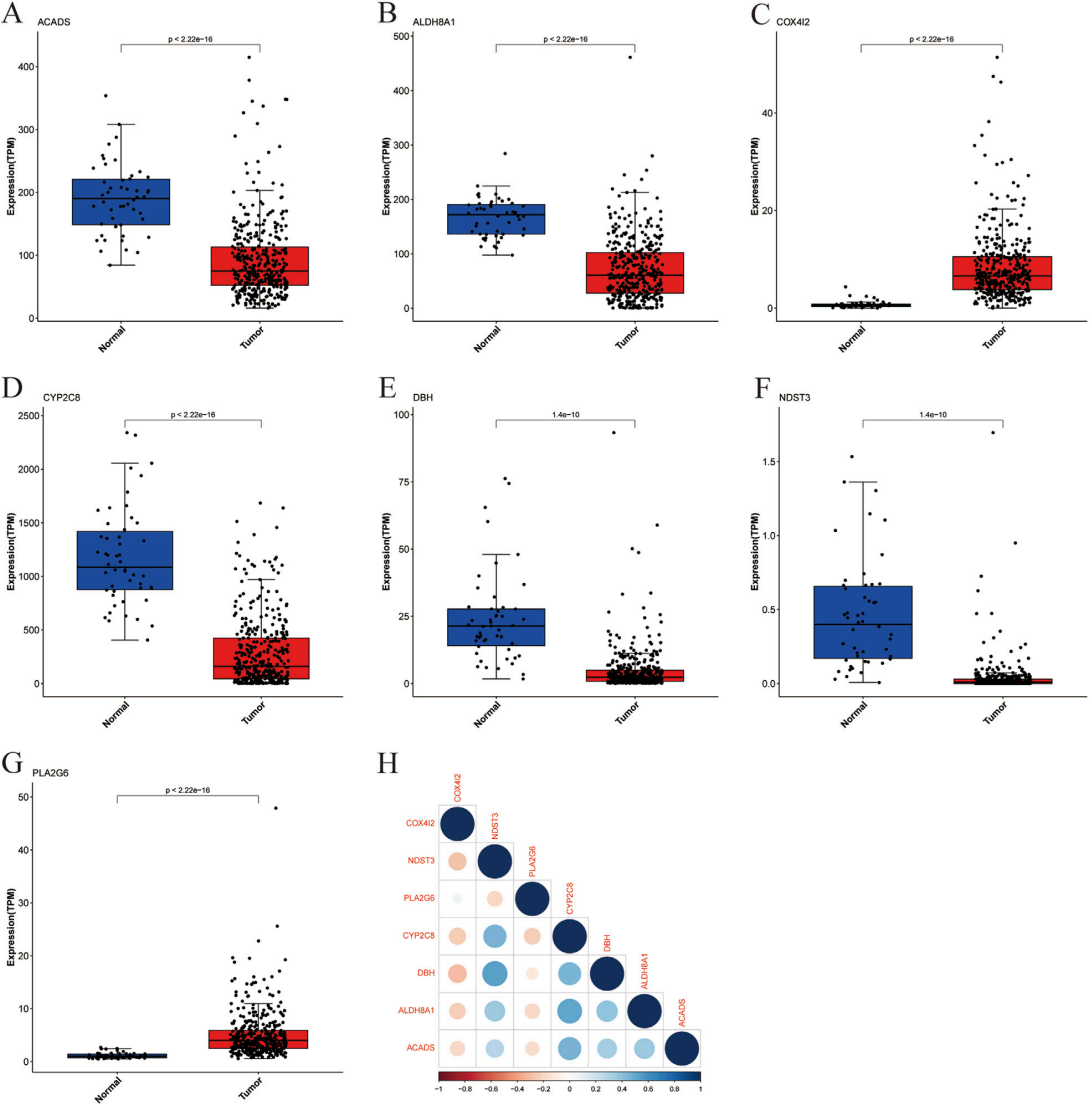
Boxplot of marker gene expression in cancer and healthy individuals with correlation analysis. **(A)** ACADS, **(B)** ALDH8A1, **(C)** COX4I2, **(D)** CYP2C8, **(E)** DHB, **(F)** NDST3, **(G)** PLA2G6, **(H)** Heatmap of Marker Gene Correlations.

We analyzed the expression patterns of the seven above-identified marker genes (ACADS, ALDH8A1, COX4I2, CYP2C8, DBH, NDST3, and PLA2G6) in both cancerous and healthy human tissues to investigate their potential involvement in liver cancer. The distribution of expression levels is shown in Figures 4A–G. The expression levels of the marker genes were significantly altered in tumor samples (represented in red) compared to the normal samples (represented in blue). ACADS (Figure 4A), ALDH8A1 (Figure 4B), CYP2C8 (Figure 4D), DBH (Figure 4E), and NDST3 (Figure 4F) showed significantly reduced expression in tumor tissues compared with those in normal tissue, suggesting their potential downregulation in liver cancer. However, COX4I2 (Figure 4C) and PLA2G6 (Figure 4G) exhibited a higher expression in tumor tissues, with a marked increase compared to normal tissues (*P* < 2.22 × 10^−16^). The correlation between the expression levels of the marker genes was evaluated through a pairwise correlation analysis, which is presented in Figure 4H.

The results showed significant correlations between several genes, highlighting potential interactions within metabolic or signaling pathways relevant to liver cancer. ALDH8A1 and CYP2C8, ACADS and CYP2C8, as well as DBH and NDST3, showed strong positive correlations, suggesting that these genes may share common regulatory mechanisms or be co-regulated in the context of liver cancer. COX4I2 exhibited a negative correlation with several other marker genes, including DBH and ALDH8A1, indicating distinct roles or opposing effects in the tumorigenic processes.

**FIGURE 5:**
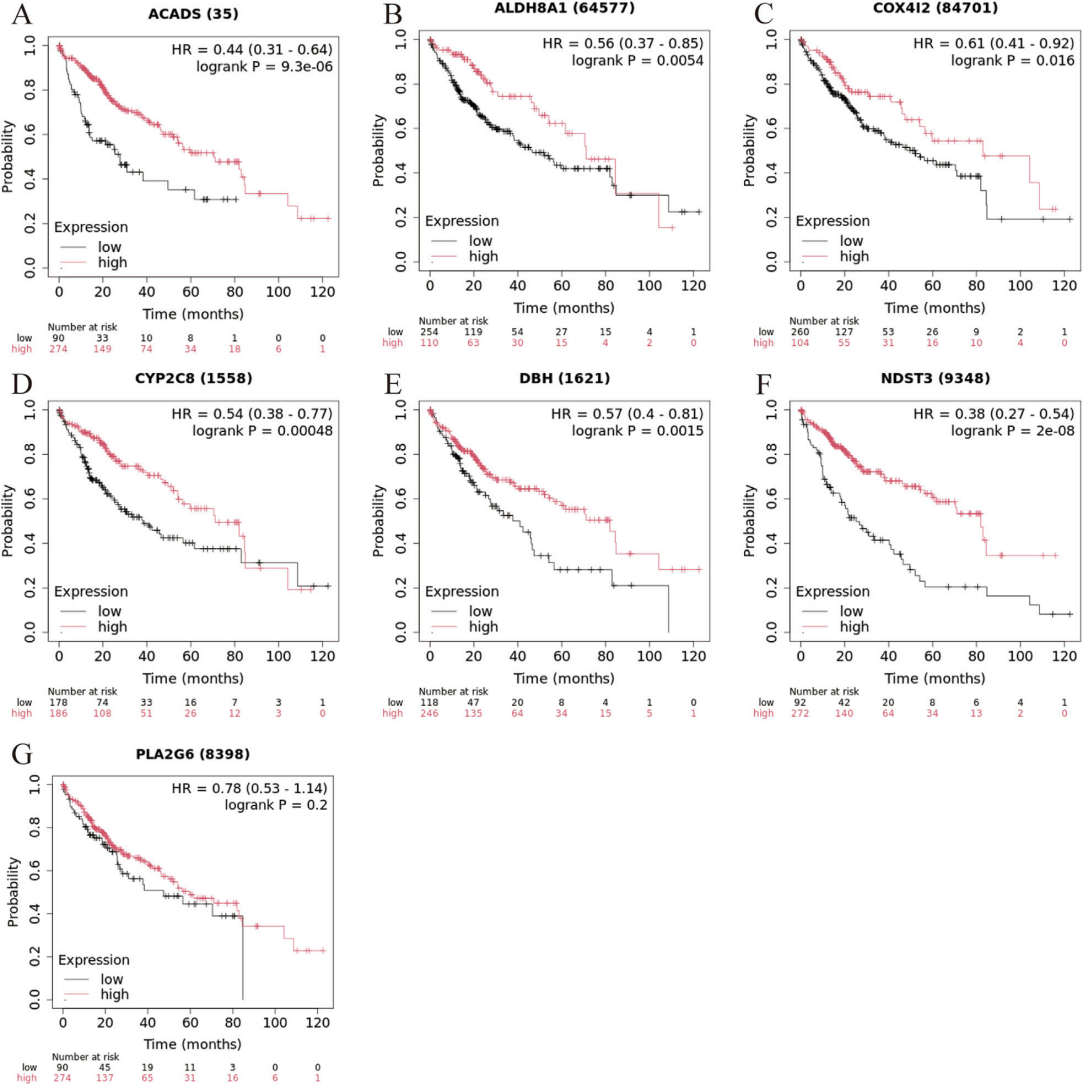
Kaplan-Meier survival curve for liver cancer based on marker gene expression levels. **(A)** ACADS, **(B)** ALDH8A1, **(C)** COX4I2, **(D)** CYP2C8; **(E)** DHB, **(F)** NDST3, **(G)** PLA2G6.

**FIGURE 6:**
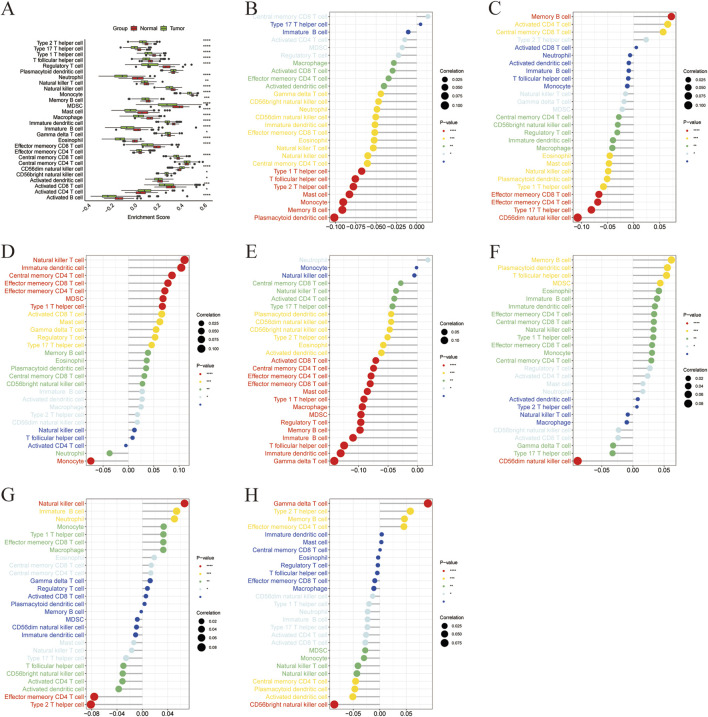
Correlation between marker gene and immune cells. **(A)** Boxplot of ssGSEA enrichment scores of immune cells in high and low expression groups of marker genes, **(B)** Correlation between ACADS and immune cells, **(C)** Correlation between ALDH8A1 and immune cells, **(D)** Correlation between COX4I2 and immune cells, **(E)** Correlation between CYP2C8 and immune cells, **(F)** Correlation between DHB and immune cells, **(G)** Correlation between NDST3 and immune cells, **(H)** Correlation between PLA2G6 and immune cells.

To evaluate the prognostic significance of the seven marker genes in liver cancer, survival analysis was performed based on the expression levels of the seven marker genes. The survival curves for each marker gene, divided into high and low expression groups, are displayed in Figure 6. High expression of ACADS (Figure 5A, HR = 0.44, 95% CI: 0.31-0.64, log-rank *P* = 9.3 × 10^−6^), ALDH8A1 (Figure 5B, HHR = 0.56, 95% CI: 0.37-0.85, log-rank *P* = 0.0054), COX4I2 (Figure 5C, HR = 0.61, 95% CI: 0.41-0.92, log-rank *P* = 0.016), CYP2C8 (Figure 5D, HR = 0.54, 95% CI: 0.38-0.77, log-rank *P* = 0.00048), DBH (Figure 5E, HR = 0.57, 95% CI: 0.40-0.81, log-rank *P* = 0.0015) and NDST3 (Figure 5F, HR = 0.38, 95% CI: 0.27-0.54, log-rank *P* = 2 × 10^−8^) were associated with significantly better survival outcomes compared to low expression, suggesting their potential as a protective factor in liver cancer. While high expression of PLA2G6 tended to improve survival, the result is not statistically significant (Figure 5G HR = 0.78, 95% CI: 0.53-1.14, log-rank *P* = 0.2), suggesting that its role in liver cancer prognosis may require further investigation.

### Pathway enrichment and immune cell correlation analysis of marker genes in liver cancer

To explore the biological pathways potentially associated with the expression of the seven marker genes in liver cancer, GSEA was performed (Supplementary Figures S2A–E). The GSEA results for ACADS (Supplementary Figure S2A) revealed significant enrichment in the fatty acid degradation pathway, as well as in fatty acid metabolism, indicating its potential involvement in lipid metabolism processes within liver cancer. Beta-Alanine metabolism was also significantly enriched, further suggesting a broader role for ACADS in metabolic reprogramming in liver cancer cells. For ALDH8A1 (Supplementary Figure S2B), significant enrichment was observed in the tryptophan metabolism pathway, highlighting its possible involvement in amino acid metabolism. For COX4I2 (Supplementary Figure S2C), significant enrichment was observed in the cardiac muscle contraction. CYP2C8 (Supplementary Figure S2D) was strongly enriched in pathways related to drug metabolism and chemical carcinogenesis. The pathway enrichment analysis for DBH (Supplementary Figure S2E) showed significant enrichment in tyrosine metabolism, indicating a potential involvement of DBH in the regulation of neurotransmitter synthesis and cellular signaling pathways, which are crucial for tumor growth and progression in liver cancer.

To investigate the potential immune-related roles of the seven identified marker genes in liver cancer, we performed a correlation analysis between marker gene expression and immune cell enrichment scores. The correlation matrix of all immune cell types is shown in Figure 6A. In general, significant correlations were observed between the expression levels of the marker genes and immune cell types, indicating that these genes may influence immune cell infiltration in the tumor microenvironment. ACADS expression showed significant positive correlations with several immune cell types (Figure 6B), particularly with Memory B cells, indicating a potential role in immune regulation. ALDH8A1 expression was strongly correlated with type 17 T helper cells and CD56dim natural killer cells (Figure 6C). The expression of COX4I2 showed significant correlations with Natural Killer T cells (Figure 6D), indicating that COX4I2 might be involved in regulating the activation of innate immune responses. CYP2C8 expression was positively correlated with immature dendritic cells and gamma delta T cells (Figure 6E). DBH expression exhibited a strong negative correlation with CD56dim natural killer cells (Figure 6F), suggesting that DBH may be involved in regulating the immune suppression in liver cancer. Lower DBH expression might be associated with reduced CD56dim natural killer cell activation, possibly affecting the anti-tumor immune response. The expression of NDST3 was positively correlated with natural killer cells and negatively correlated with effector memory CD4 T cells and type 2 T helper cells (Figure 6G). PLA2G6 expression showed a significant positive correlation with gamma delta T cells and a strong negative correlation with CD56bright natural killer cells (Figure 6H).

### Gene-gene-drug interaction network

**FIGURE 7:**
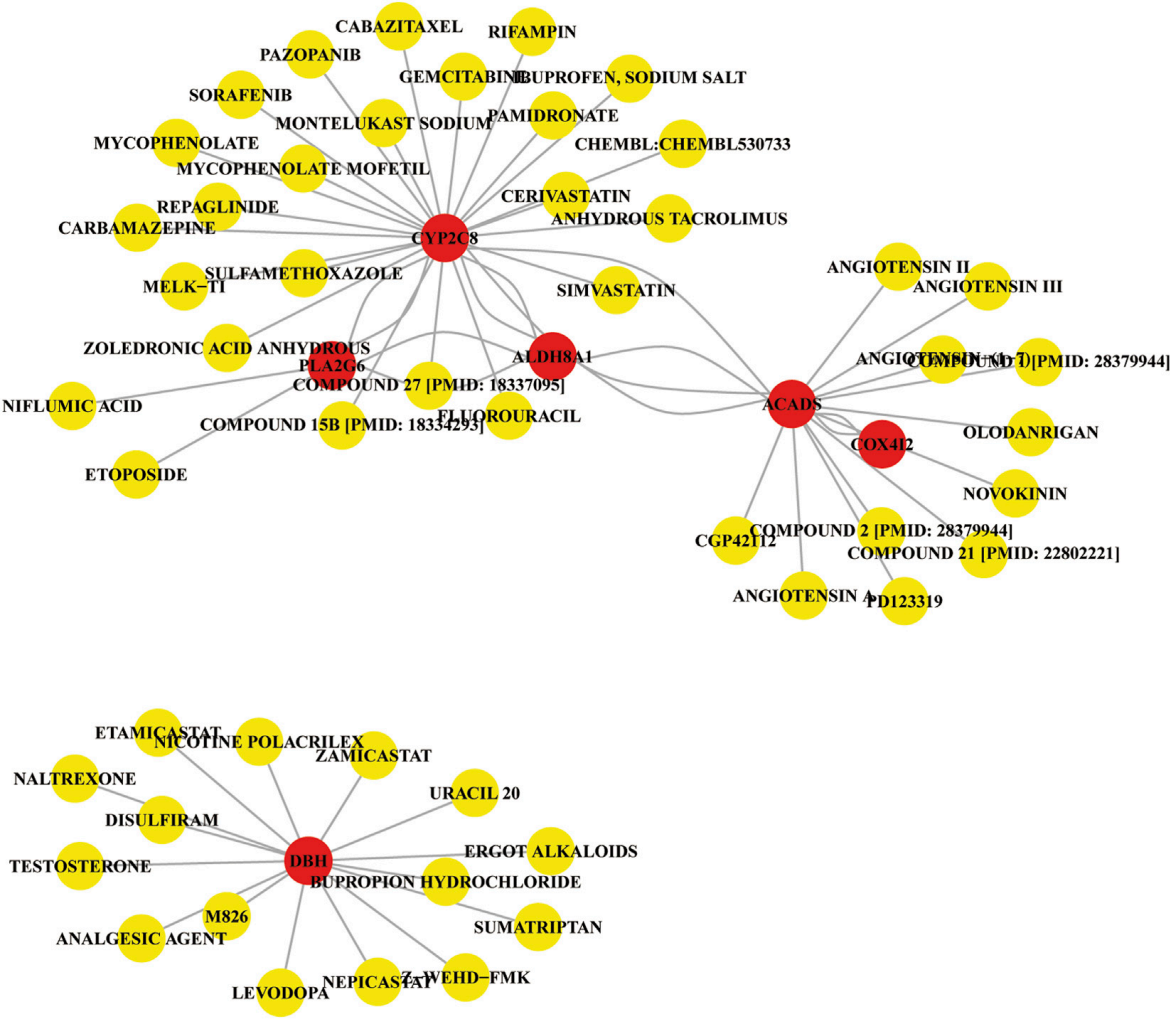
Gene-gene-drug interaction network Diagram.

To investigate the potential therapeutic implications of the identified marker genes in liver cancer, we constructed a gene-gene-drug interaction network based on the PPI analysis (Protein-Protein Interaction) and drug-gene interactions obtained from the DGIdb v4.0 database. The resulting network (Figure 7) integrates the interactions between the marker genes and several potential therapeutic drugs. The PPI analysis identified several key gene-gene interactions within the liver cancer context. These interactions suggest that the marker genes may collaborate in regulating metabolic pathways and immune responses relevant to tumor progression. For example, CYP2C8, ALDH8A1, and ACADS were shown to interact with proteins involved in drug metabolism and oxidative stress, indicating their critical roles in liver cancer metabolism and detoxification.

The drug-gene interaction network revealed several drugs that target the identified marker genes or their associated proteins. Some notable drug interactions include: Sorafenib, a frontline therapy for liver cancer, interacts with CYP2C8, indicating that it may influence metabolic reprogramming in liver cancer cells. Compound 27 was found to interact with PLA2G6, CYP2C8, and ALDH8A1, pointing to its potential in regulating immune responses and the metabolic balance, and may be useful for liver cancer treatment. Other drugs such as pazopanib, etoposide and mycophenolate mofetil also showed interactions with the marker genes, indicating possible therapeutic repurposing opportunities for liver cancer treatment. The gene-gene-drug interaction network provides a visual representation of the relationships between the identified marker genes, their protein interactions, and the potential drugs that target these genes or their interacting proteins. The network highlights several drugs that may be useful for liver cancer treatment by targeting key molecular pathways involved in metabolic reprogramming, immune modulation, and tumor progression. Drugs like sorafenib, simvastatin, and gemcitabine are already in clinical use for liver cancer and show strong interactions with key marker genes, reinforcing their therapeutic potential.

### Validation of the marker genes’ expression in liver cancer and an external dataset

**FIGURE 8:**
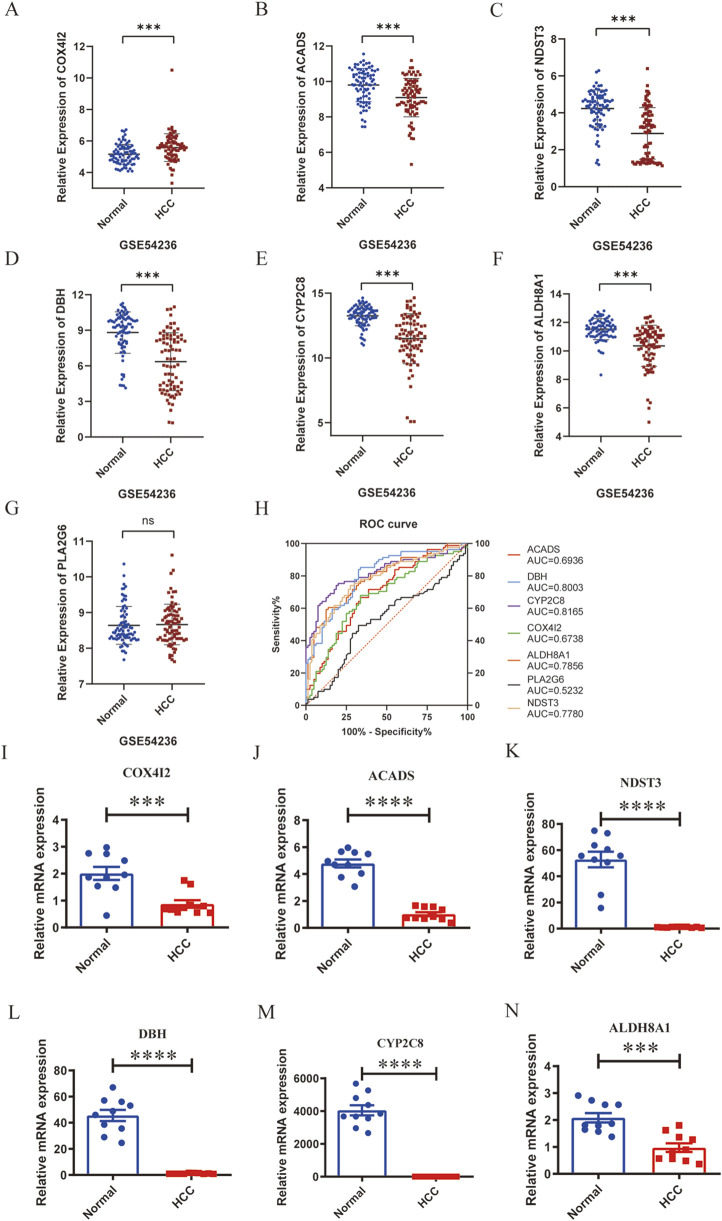
Validation of the marker genes’ expression in liver cancer and an external dataset. A-G, Relative expression levels of COX4I2 **(A)**, ACADS **(B)**, NDST3 **(C)**, DHB **(D)**, CYP2C8 **(E)**, ALDH8A1 **(F)**, and PLA2G6 **(G)** in the dataset of GSE54236. **(H)**, ROC analysis of COX4I2, ACADS, NDST3, DHB, CYP2C8, ALDH8A1, and PLA2G6 in the dataset of GSE54236. I-N, Relative mRNA expression of COX4I2 **(I)**, ACADS **(J)**, NDST3 **(K)**, DHB **(L)**, CYP2C8 **(M)**, and ALDH8A1 **(N)**, n = 10 per group, triplicate technical replicates, *p < 0.05, **p < 0.01, ***p < 0.001, ****p < 0.0001.

To validate the transcriptional patterns of the metabolism-related marker genes (ACADS, ALDH8A1, COX4I2, CYP2C8, DBH, NDST3, and PLA2G6), we firstly examined the expression levels of these seven genes in an external dataset GSE54236. Similar to TCGA hepatic carcinoma cohort data, the results demonstrated that ACADS, ALDH8A1, CYP2C8, DBH, and NDST3 were significantly downregulated, and COX4I2 was upregulated, while PLA2G6 showed no significant change in liver cancer tissues compared with those in normal tissues (Figures 8A–G). Furthermore, ROC analysis revealed ACADS, ALDH8A1, COX4I2, CYP2C8, DBH, and NDST3 possessed good ability to predict the occurrence of liver cancer, with AUC value of 0.6936 for ACADS, 0.7856 for ALDH8A1, 0.6738 for COX4I2, 0.8165 for CYP2C8, 0.8003 for DBH, and 0.7780 for NDST3 (Figure 8H). Additionally, we collected 10 paired tumor tissues and adjacent non-tumorous tissues from liver cancer patients. RT-PCR assays confirmed significant downregulation of ACADS (95% CI: 4.469 to −3.076), ALDH8A1 (95% CI: 1.611 to −0.6145), COX4I2 (95% CI: 1.719 to −0.5509), CYP2C8 (95% CI: 4690 to −3399), DBH (95% CI: 53.38 to −35.47), and NDST3 (95% CI: 64.31 to −39.26) in liver cancer tissues compared with those in the matched peri-tumoral controls (all *P* < 0.05) (Figures 8I–N). These results confirmed that the expression levels of ACADS, ALDH8A1, COX4I2, CYP2C8, DBH, and NDST3 were decreased in liver cancer tissues.

## Discussion

In this study, we collected transcriptional profiles of liver cancer, a disease known for its associated metabolic reprogramming. By integrating transcriptomic data from the TCGA database with WGCNA, we identified 234 metabolism-related differentially expressed genes (DEGs). Through machine learning algorithms, we further refined the list and identified seven key metabolism-related marker genes-ACADS, ALDH8A1, COX4I2, CYP2C8, DBH, NDST3, and PLA2G6. These genes were significantly associated with patient survival and immune cell infiltration, highlighting their potential as prognostic biomarkers. Notably, the majority of these genes demonstrated strong prognostic value, with ACADS, ALDH8A1, CYP2C8, DBH, and NDST3 emerging as protective factors, while PLA2G6’s expression level did not independently correlate with overall survival. Actually, differential expression analysis compared tumor vs. normal tissues, reflecting cancer-associated dysregulation. Prognostic analysis assessed inter-tumor heterogeneity among cancer patients. PLA2G6 expression might not stratify overall survival if its variation is similar across patients with liver cancer. Additionally, its survival impact might be compensated by parallel pathways. And the expressions of ACADS, ALDH8A1, COX4I2, CYP2C8, DBH, and NDST3 were confirmed in the tissues of patients with liver cancer and an external dataset GSE54236. Our study unveiled the metabolism-related marker genes in liver cancer by using machine learning, which were not investigated before. Additionally, we identified the candidate drugs which would target these marker genes, providing therapeutic strategies for liver cancer treatment.

This study depends on the integration of multiple bioinformatics approaches, including WGCNA, machine learning, and gene-drug interaction network analysis, to pinpoint metabolic genes that could serve as new therapeutic targets. Unlike previous studies that often focus on individual metabolic pathways or specific gene targets, our approach offers a comprehensive multi-dimensional analysis of metabolism in liver cancer, providing deeper insights into the underlying molecular mechanisms. Furthermore, we established a robust link between these genes and immune cell infiltration, an area that remains underexplored in liver cancer research. The identification of potential therapeutic drugs, such as Pazopanib, Sumatriptan, and Etoposide, provides promising avenues for repurposing existing drugs in the context of liver cancer treatment. The integrative WGCNA and tri-modal machine learning (RF/SVM/LASSO) framework represents a core methodological advance (Shi et al., 2025). While WGCNA identifies phenotype-associated gene modules, it may retain non-specific genes. Sequential machine learning application refines hub genes through tri-algorithm consensus, mitigating model-specific biases. Only genes selected by all three algorithms are retained, ensuring robust, anti-overfitting candidates.

In this study, we identified several marker genes (ACADS, ALDH8A1, COX4I2, CYP2C8, DBH, and NDST3) as potential prognostic biomarkers for liver cancer. The expression profiles of these genes in both tumor and normal tissues revealed distinct alterations that may offer insights into their roles in the metabolic reprogramming of liver cancer and their potential as therapeutic targets. ACADS, which showed significantly reduced expression in liver cancer tissues, is an enzyme involved in the mitochondrial fatty acid β-oxidation pathway (Ding et al., 2022). The downregulation of ACADS in liver cancer may suggest a shift away from fatty acid oxidation towards other metabolic pathways, such as glycolysis or lipid biosynthesis, which are commonly upregulated in cancers to support rapid cell proliferation (Gu et al., 2025; Wang et al., 2022). Additionally, ACADS was associated with carbohydrate metabolism in liver cancer (Huang et al., 2024). The loss of ACADS could thus be a contributing factor to the altered metabolic state of liver cancer cells. ALDH8A1, which is also downregulated in liver cancer tissues, is involved in the oxidative metabolism of aldehydes, including fatty aldehydes (Liu et al., 2024). Similar to ACADS, its reduced expression might reflect a metabolic shift away from oxidative pathways and suggest an accumulation of toxic metabolites in liver cancer. The downregulation of both ALDH8A1 and ACADS further supports the hypothesis that liver cancer cells adapt their metabolism to evade cell death mechanisms and support their rapid growth. COX4I2 is a hypoxia related gene that is associated with poor prognosis and progression of various cancers by regulating the respiratory chain. In liver cancer, overexpression of HIF-1α and HIF-2α is consistently observed, and this phenomenon is linked to unfavorable clinical prognoses (Wong et al., 2014). Specifically, HIF-1 reduces reduced electron transfer efficiency across the electron transport chain by upregulating NDUFA4L2 and COX4I2. This adaptive metabolic reprogramming serves as a protective mechanism to mitigate excessive reactive oxygen species (ROS) accumulation, thereby promoting tumor cell survival under hypoxic conditions (Fukuda et al., 2007). This hypothesis requires experimental verification. CYP2C8, which showed lower expression in liver cancer tissues, is involved in the metabolism of a variety of endogenous and exogenous compounds, including drugs and fatty acids (Manhas, 2023; Yue and Hirao, 2023). And it served as a fatty acid metabolism-related biomarker in liver cancer (Zhao et al., 2024). CYP2C8 has been linked to both carcinogenesis and resistance to chemotherapy, making it a potential therapeutic target for overcoming drug resistance in liver cancer treatment. DBH, an enzyme responsible for the conversion of dopamine to norepinephrine, was also significantly downregulated in liver cancer. This suggests that neurotransmitter regulation may play a role in liver cancer progression. Recent studies have shown that neurotransmitters such as dopamine and norepinephrine can influence tumor microenvironments and promote cancer cell survival. The reduced expression of DBH (Bao et al., 2018) in liver cancer may lead to dysregulation of the adrenergic system, which is involved in cellular responses to stress, inflammation, and immune evasion. The decreased expression of NDST3 could impact the tumor microenvironment and affect key signaling pathways, such as those related to growth factors and the extracellular matrix, which are critical for tumor progression and metastasis (Zhang Z. et al., 2019). Its downregulation may contribute to changes in cell-cell communication and promote an aggressive phenotype in liver cancer cells. In the future, we can investigate the transcription factors and regulatory pathways of these genes through the SE database (Qian et al., 2023), so as to more effectively reveal their functions.

The correlation analysis of these marker genes revealed strong positive correlations between certain genes, such as ALDH8A1 and CYP2C8, and between ACADS and CYP2C8, suggesting that these genes may be co-regulated and functionally interconnected within metabolic or signaling pathways relevant to liver cancer. These correlations may provide new insights into the underlying mechanisms that drive liver cancer metabolism and offer novel therapeutic targets for future clinical interventions. On the other hand, the negative correlations observed between COX4I2 and other marker genes, such as DBH and ALDH8A1, highlight the complexity of the metabolic reprogramming in liver cancer and suggest that these genes might play distinct or even opposing roles in the tumorigenic processes.

Our study revealed significant correlations between the expression levels of the marker genes and immune cell types, including T cells, B cells, macrophages, and NK cells. The immune microenvironment was critical for the defense against tumor cells. Previous research reported that M1 macrophages and cytotoxic T lymphocytes played an anti-tumor role in the progression of liver cancer (Cheng et al., 2022). The current immunotherapeutic approaches mainly target against cytotoxic T lymphocytes (Kudo, 2019). In addition, type I NK cells would inhibit the development of liver cancer via pro-inflammatory response (Oura et al., 2021). Interestingly, we found that ACADS, ALDH8A1, CYP2C8, DBH, and NDST3 were significantly correlated with the infiltration of T cells, NK cells, and macrophages. However, the expressions of these marker genes were downregulated in liver cancer, indicating that a decrease in these marker genes may promote the progression of liver cancer by attenuating the immune response.

Then the gene-gene-drug interaction network was explored to identify drugs targeting the marker genes and their associated proteins, revealing potential therapeutic opportunities for liver cancer treatment. Previous study revealed that Gamabufotalin, a compound derived from traditional Chinese medicine, inhibited liver cancer progression by modulating metabolic pathways through STAMBPL1 downregulation (Zheng et al., 2024). The activity of CYP2C could be mediated by Oridonin (Yi-Wen et al., 2018). Notably, we found that sorafenib, a frontline therapy for liver cancer, interacts with CYP2C8, suggesting that it may influence metabolic reprogramming in liver cancer cells. Sorafenib is widely used in clinical practice for hepatocellular carcinoma, and its effects extend beyond inhibiting tumor angiogenesis, potentially enhancing its anticancer effects through modulation of metabolic pathways. Additionally, a compound (compound 27) was found to interact with PLA2G6, CYP2C8, and ALDH8A1, highlighting its potential in regulating immune responses and maintaining metabolic balance, which may be valuable for liver cancer treatment. These interactions provide a theoretical foundation for developing new therapeutic strategies targeting immune modulation and metabolic reprogramming pathways that promote tumor progression. Other drugs, such as pazopanib, etoposide, and mycophenolate mofetil, also showed interactions with the marker genes, suggesting potential repurposing for liver cancer therapy. These drugs may exert their therapeutic effects by altering metabolic pathways, modulating immune responses, or regulating the tumor microenvironment to inhibit cancer cell growth or overcome drug resistance.

The gene-gene-drug interaction network provides a visual representation of the relationships between the identified marker genes, their interacting proteins, and the potential drugs targeting these genes or their associated proteins. Drugs like sorafenib, simvastatin, and gemcitabine, which are already in clinical use for liver cancer, exhibit strong interactions with key marker genes, further reinforcing their therapeutic potential. Our findings not only deepen the understanding of metabolic reprogramming, immune modulation, and tumor progression pathways in liver cancer but also offer important insights into the development of novel therapeutic strategies and drug candidates for liver cancer treatment.

### Limitation

Even though our study identified seven metabolism-related marker DEGs in liver cancer, among them, six genes (ACADS, ALDH8A1, COX4I2, CYP2C8, DBH, NDST3) could predict the prognosis of liver cancer, the sample type was simple and only obtained from the TCGA database. In addition, even though we have confirmed the expression of these six genes in liver cancer tissues, the effects of these marker genes on the progress of liver cancer should further confirm by experiments. Furthermore, while machine learning accelerates the discovery of biomarkers by processing high-dimensional data, machine learning models are often “black boxes”—their decision-making processes are opaque. Moreover, most machine learning models treat features as static entities, failing to account for temporal or conditional variations. This limits the ability to identify context-specific biomarkers, which are crucial for personalized medicine. Consequently, rigorous biological validation remains imperative.

## Conclusion

In summary, our study provides a comprehensive bioinformatics analysis to identify ACADS, ALDH8A1, COX4I2, CYP2C8, DBH, and NDST3 as key metabolism-related marker genes in liver cancer. These genes demonstrate multidimensional involvement in metabolic reprogramming and immune microenvironment modulation, suggesting their dual potential as prognostic indicators and therapeutic targets. In addition, the identification of candidate drugs, such as Pazopanib and Sumatriptan, offers exciting possibilities for the repurposing of existing drugs and the development of new therapies for liver cancer. However, future studies must validate their biological relevance through *in vivo* functional assays to confirm causal roles in tumor progression, mechanistic studies elucidating how these genes affect liver cancer development, and evaluation the efficiency of candidate drugs in animal models. While our findings illuminate novel therapeutic avenues targeting metabolic-immunological synergies, translating this potential into clinical applications necessitates rigorous functional validation.

## Data Availability

Publicly available datasets were analyzed in this study. This data can be found in the TCGA database https://portal.gdc.cancer.gov via the TCGA-Liver Cancer dataset; and here: https://www.ncbi.nlm.nih.gov/geo/ accession number GSE54236.
