# Supplementary material for: Identifying metabolism-related genes in liver cancer through weighted gene co-expression network analysis and machine learning
Source: Front Genet. 2025 Sep 24;16:1654459. doi: 10.3389/fgene.2025.1654459 (PMC12504094; doi:10.3389/fgene.2025.1654459)
Supplement: Supplementary file 3 [file Table1.docx]

| **Supplementary Table 1. Primers used in the manuscript.** | | |
| --- | --- | --- |
| **Gene name** | **Forward primers** | **Reverse primers** |
| ACADS | CGGCAGTTACACACCATCTAC | GCAATGGGAAACAACTCCTTCTC |
| ALDH8A1 | CTGAACCAGGTGGCGGATTT | CCTGGTGGAACACCGACTC |
| COX4I2 | ACTACCCCATGCCAGAAGAG | TCATTGGAGCGACGGTTCATC |
| CYP2C8 | TCTTTCACCAATTTCTCAAAAGTCT | CCATCCCAAAATTCCGCAAGG |
| DBH | TCACCTCCTGCACGTACAAC | TGAACTGCTGAGACACGGAC |
| NDST3 | GACCCTGCAGCTCTGAAGTT | TCTCGATGTGGCTGGCATAC |
| β-actin | ATCTGGCACCACACCTTC | AGCCAGGTCCAGACGCA |
