# Supplementary material for: Identifying metabolism-related genes in liver cancer through weighted gene co-expression network analysis and machine learning
Source: Front Genet. 2025 Sep 24;16:1654459. doi: 10.3389/fgene.2025.1654459 (PMC12504094; doi:10.3389/fgene.2025.1654459)
Supplement: Supplementary file 5 [file Table5.docx]

| **Supplementary Table 5. Metrics of the three machine learning models.** | | | | | | |
| --- | --- | --- | --- | --- | --- | --- |
| **Model** | **Accuracy** | **Sensitivity** | **Specificity** | **Precision** | **F1** | **AUC** |
| LASSO | 0.9609375 | 0.9732143 | 0.875 | 0.981982 | 0.9775785 | 0.9916295 |
| SVM | 0.9910714 | 0.875 | 0.9823009 | 0.9453125 | 0.9866667 | 0.9955357 |
| RF | 0.9910714 | 0.9375 | 0.9910714 | 0.9910714 | 0.9955357 | 0.984375 |
