# Supplementary material for: Identifying metabolism-related genes in liver cancer through weighted gene co-expression network analysis and machine learning
Source: Front Genet. 2025 Sep 24;16:1654459. doi: 10.3389/fgene.2025.1654459 (PMC12504094; doi:10.3389/fgene.2025.1654459)
Supplement: Supplementary file 6 [file DataSheet1.docx]

**Supplementary Figures and figure legends**


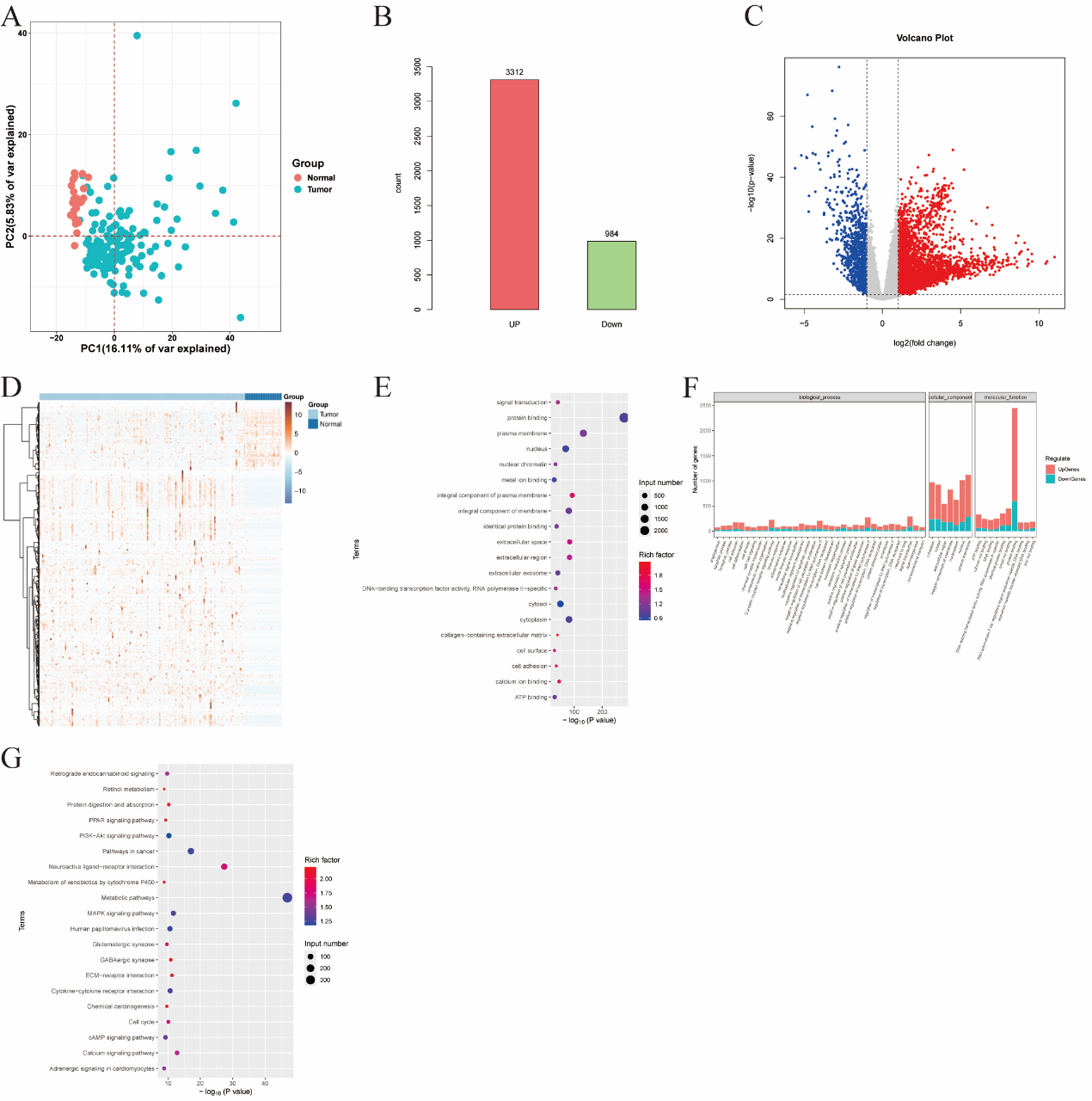


**Supplementary Figure 1. Identification of the DEGs in liver cancer.** A: PCA analysis of samples in the tumor or normal groups. B: The count of DEGs in tumor or normal groups. C: Volcano plot of DEGs. D: Heat map of DEGs. E: GO analysis of DEGs. F: The BP, MF, and CC results of GO analysis. G: KEGG analysis of DEGs.


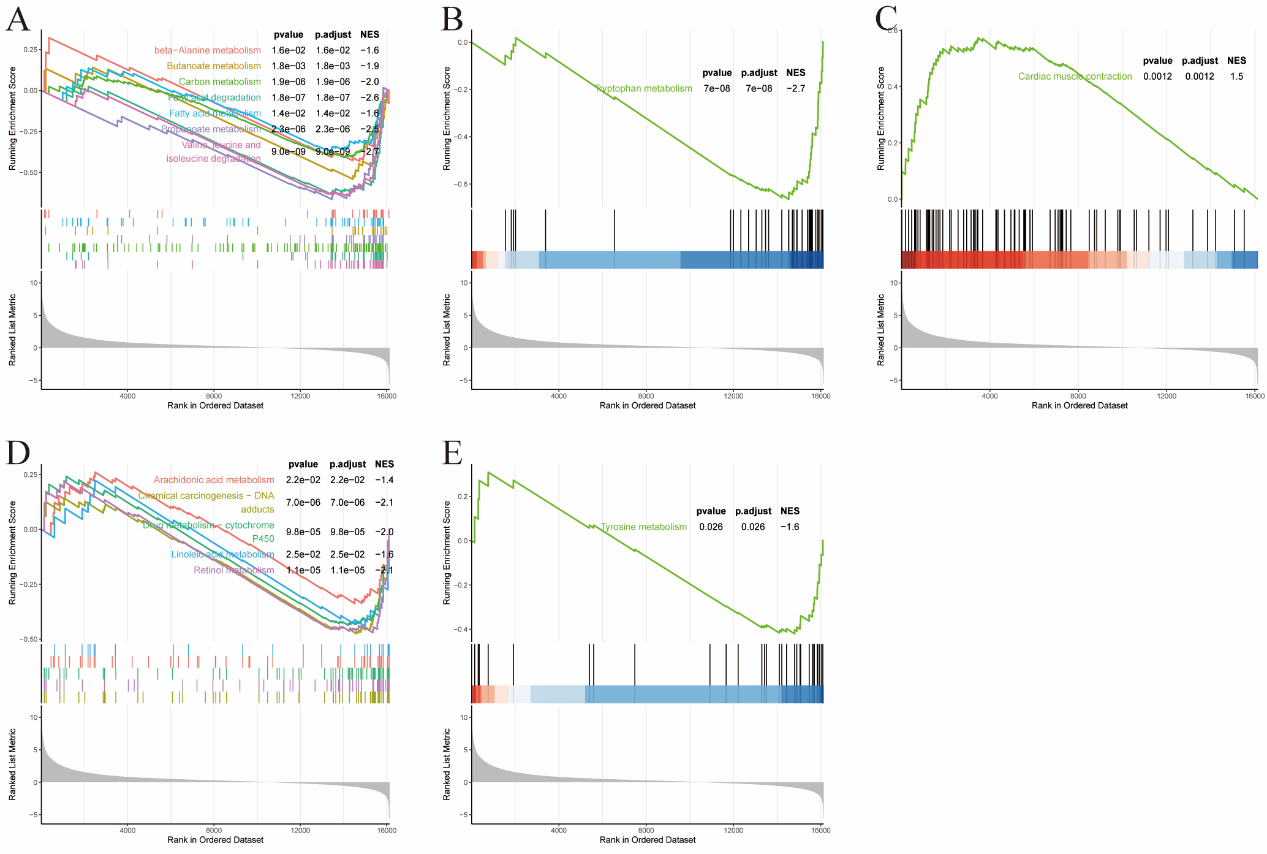


**Supplementary Figure 2. GSEA enrichment plot of pathways involved by marker Genes.** A: By ACADS, B: By ALDH8A1, C: By COX4I2, D: By CYP2C8; E: By DHB.
